# Parents’ concerns regarding the growth characteristics of their adolescents: a qualitative inquiry in Iran

**DOI:** 10.1080/17482631.2018.1453179

**Published:** 2018-04-12

**Authors:** Mohammad Ali Cheraghi, Parvaneh Rezasoltani, AbouAli Vedadhir, Ziba Taghizadeh, Seyyed Hossein Samadanifard

**Affiliations:** a Department of Nursing, School of Nursing & Midwifery, Tehran University of Medical Sciences, Tehran, Iran; b PhD candidate, Department of Reproductive Health & Midwifery, School of Nursing & Midwifery, Tehran University of Medical Sciences, Tehran, Iran; c Department of Midwifery, School of Nursing and Midwifery, Guilan University of Medical Sciences, Rasht, Iran; d Department of Anthropology, School of Social Sciences, University of Tehran, Tehran, Iran; e UCL Department of Science and Technology Studies (STS), University College London, London, UK; f Department of Reproductive Health & Midwifery, School of Nursing & Midwifery, Tehran University of Medical Sciences, Tehran, Iran; g Department of Endocrinology, Hazrat-e Rasool General Hospital, School of Medicine, Iran University of Medical Sciences, Tehran, Iran

**Keywords:** Parents, concern, adolescent, growth, constructivist grounded theory

## Abstract

In recent times, parents have become increasingly concerned, both subjectively and objectively, about their adolescents' body height/weight growth. Parent-adolescent interactions about this issue and the potential socio-psychological consequences of such interactions should be considered as an important influencing factor on the future of adolescents' sexual and reproductive health. To achieve a greater understanding of such concerns, it is necessary to further elucidate parents' experiences on this topic, so as to expand the existing literature. This study aimed to explain the perceptions of parents' concerns regarding their adolescents' growth characteristics in the socio-cultural context of Iran as a transitional society. This paper is part of a larger qualitative study designed using the Constructivist Grounded Theory Methodology (CGTM). We conducted open-ended intensive interviews with eleven parents individually and recruited them through purposeful and theoretical sampling from a teaching hospital, community, and a primary school in Tehran with theoretical sampling variation in terms of teenagers' age, sex, and birth order, place of residence, parents' occupation and education, and the self-reported socio-economic status. Using the analytical procedures of the CGTM, we performed analyses. In the findings, the concept of 'living with constant sense of uncertainty' emerged from the subcategories including 'feeling existing and potential concern about expected minimum and maximum bio-positions of growth,' 'feeling potential concern about biological health consequences,' 'feeling potential concern about the emergence of early/late maturity signs,' 'feeling potential concern about adolescent's emotional threat,' 'feeling concerned about future employment, education, marriage, and fertility,' and 'feeling potential concern about the society's view'. These findings suggest that parents are living with a constant sense of uncertainty about their teens' growth characteristics throughout the transition from adolescence. All stakeholders including parents, health-care practitioners and policymakers, and anthropologists/sociologists should be focus on such concerns, in order to manage them and their possible socio-psychological burdens.

## Introduction

The World Health Organization defines adolescence as the period between childhood and adulthood, age from 10 to 19 years. It is the start of reproductive age (Hills & Byrne, 2010; World Health Organization [WHO], 2017). Some distinct aspects of this period include biological processes such as considerable pace in body height and weight growth, body composition, and the development of the reproductive and sexual system. These characteristic definitions are affected by the socio-emotional processes in different societies in the world (Jeddi et al., 2014; WHO, 2017; Yousefi et al., 2013).

Adolescents have been the centre of attention in health-related planning including Millennium Development Goals worldwide (Sawyer et al., 2012). One component of primary health care (PHC) is the monitoring of height and weight growth in adolescence, through the use of growth standards and anthropometric measurements. Based on the growth curves, the incompatibility of healthy adolescents’ height/weight growth in comparison with the average in their peers may evoke the concern of parents (Rogol & Hayden, 2014). It is important for some parents to have the adult height of their children in pre-/early adolescence predicted by experts and, if necessary, they should try to increase their height (Carel, 2004; Soliman, De Sanctis, Elalaily, & Bedair, 2014).

In some cases, these concerns may be rooted in the parents’ understanding or perceptions. They may be more concerned about the difference in the height/weight of adolescents compared to those of their peers because it may have an effect on the adolescent’s emotional, social, sexual and reproductive performances and consequences. (Case & Paxson, 2008; Cheung et al., 2013; Deaton & Arora, 2009; Lee et al., 2009; Magnusson et al., 2005; Rees, Sabia, & Argys, 2009; Samaras, 2012; Balen, Sinnema, & Geenen, 2006; Balen et al., 2005). However, these associations could be related to other stressful stimuli (Bjerkeset, Romundstad, Evans, & Gunnell, 2008; Kelnar, 2012).

As proposed by a group of Australian researchers in a structured cross-sectional study (2008), clinical and lay definitions of the terms “short/tall height” or “excessive/light weight” may differ. In other words, the view of parents or the public on normal or abnormal height/weight growth could be a potential factor for parents’ concern about the general health of their children. The parents’ concern could be more about the damaging effect on current or future performance of their children or negative effects on their relationship with their peers rather than more/less height/weight growth of their child/adolescent. Some evidences indicate that the extreme concern of parents and their direct comments/interpretations about weight could be the reason why some girls overeat in the future, lack respect for their body, and experience more weight-gain and concern about their body shape. In this regard, low or high concerns of parents about their children’s height/weight growth could have adverse effects on their children’s or adolescent’s health and well-being. Hence, other than solutions for resolving the problem of weight/height growth, if necessary from the biomedical view, it is important to explore such concerns by a holistic approach to administer parents’ interactions/actions in coping with them in order to enhance the overall health and quality of life of their adolescents (Lampard et al., 2008)

The interaction between parents (as proximal social determinants of health) and adolescents, and parents’ subjective norms/values and their action on the growth characteristics of adolescents can positively/negatively influence their general health and well-being (Blum et al., 2012; Lindqvist, Kostenius, Gard, & Rutberg, 2015; Sawyer et al., 2012; Viner et al., 2012; Umberson, Crosnoe, & Reczek, 2010). In fact, from some parents, the most common criticisms are related to the adolescent’s physical appearance or body size and they make every effort to adjust it. Social pressures/messages that come from parents, and even other family members, and peers regarding the cultural meanings of body fitness/beauty may affect the body-image of adolescents (Chen & Jackson, 2009; Fergosen, 2011; Lindelof, Nielsen, & Pedersen, 2010; Pope, Corona, & Belgrave, 2014). Labels like “not having normal physical growth” in early/late adolescence may lead to unfavourable socio-emotional health consequences, including disparaging and reducing the person from a normal person to a defective/abnormal one, decreasing and spoiling self-confidence and social identity, and causing the person to feel discrimination (Lewis et al., 2011; Puhl, Suh, & Li, 2017).

A review of the existing literature and electronic reports in Iran regarding children’s height/weight growth revealed that most of these narrations come from biomedical models and physicians’ experience (gained in endocrine clinics) of the existence of parents’ concerns about the body height/weight of their children/adolescents or sometimes body size concerns among children/adolescents (Garousi, 2014; Mashreghi & Bahreini, 2006; Peyman, Rastegar, Taghipour, & Esmaily, 2012). Accordingly, it appears that there is a misunderstanding in some families about children’s height/weight in comparison with their peers based on assumed medical standards and social values or images. For instance, some parents prefer to have tall adolescents/children. Hence, they may try to get assistance from a physician to use hormone/medication to increase the height of their children. They consider being tall in height as an advantage in their society (Mashreghi & Bahreini, 2006). In some societies, tall people have more leadership ability and seem more attractive and beautiful. Moreover, being tall in height is positively associated with personal efficacy, income/wage, success in the job market and marriage (Balen, Sinnema, & Geenen, 2006; Case & Paxson, 2008; Balen et al., 2005), higher social position, and being stronger and more intelligent (Samaras, 2012).

In addition, Iranian researchers in the field of social sciences have asserted that most families usually relate physical beauty to social acceptance level. Some young Iranians believe that beautification of the body is against the will of God; this is from a traditional view of beauty, while modern beliefs suppose that there is no discrepancy between them. Modern standards/schemas of body size fitness as one beauty feature have encouraged many young people’s efforts to increase/decrease height, to lose weight, or to use medication to gain weight. This might be an influencing factor on the valuing of both sexes in practices such as marriage. Hence, there is an ever-increasing significance in relation to body appearance management based on body-image, particularly among young people and women in the context of Iran’s cultural transition to modernity (Charvadeh & Kermani, 2010; Zokaei, 2009).

Most qualitative studies have described adolescents/adults’ experiences of obesity and body-image, and also adolescents’ perception of their body appearance and obesity and the same perception or views by the mothers/parents (Calzo et al., 2012; Lindelof et al., 2010; McCabe et al., 2011; Peyman et al., 2012; Pope et al., 2014; Sand, Emaus, & Lian, 2015; Shrewsbury et al., 2010; Spiller, 2009; Watt & Ricciardelli, 2012; Wills et al., 2006); also, a small number of studies have addressed the issue of height (Pope et al., 2014; Watt & Ricciardelli, 2012; Wills et al., 2006). The present study attempted to conduct a qualitative inquiry to understand the dimensions of an adolescent’s height/weight growth-based concerns among Iranian parents’ perspectives. No qualitative study has been conducted on this issue from the parents’ view yet.

Social constructivists believe that a problem is not merely a harmful situation, but anything that may turn to a topic of concern among the public and politicians (Best, 2013). From this point of view, the perception/sensitivity of some Iranian parents regarding the characteristics of their children’s growth throughout adolescence as a problem can be a topic of concern. The United Nations Population Fund refers to a wide range of factors that can have an influence on adolescents’ sexual and reproductive health (United Nations Population Fund, 2016). Parents contemplating this issue and their interactions in response to it, and its potential socio-psychological consequences can be considered as one of these factors. It has been declared that focusing on socio-emotional health is fundamental to the promotion of adolescent health (Blum et al., 2012; Viner et al., 2012). Based on these viewpoints, parents, schools, health-care staff, and other stakeholders are assumed responsible for facilitating adolescents’ successful transition to adulthood (Neumark-Stainer, 2015; WHO, 2017). Focus should be on the aspects of such constructing concerns in order to modify them and manage their potential socio-psychological burdens. A national document in Iran on a comprehensive health approach (Ministry of Health, Treatment and Medical Education, Iran, 2013) indicates that reproductive and sexual health (RSH) specialists can play a major role in managing such concerns and promote the future of adolescents’ RSH.

In Iran, given the lack of a sufficient body of knowledge and literature as regards explanations of parents’ concerns about the physical growth of their adolescent, the purpose of this article was to explain the properties/dimensions of concepts connected with parents’ concerns/issues embedded in the physical growth of their adolescents which points to height/weight growth in the sociocultural context of Iran as a developing society. The authors hope to expand the existing knowledge and literature by explaining such concerns among the parents in their context.

## Methods

This study is part of the second authors' PhD thesis project in the field of RSH, using the Constructivist Grounded Theory Methodology (CGTM), developed by Kathy Charmaz (2006, 2014). We collected and analysed data using the analytical procedures of the CGTM. This genre of Grounded Theory Methodology is a social scientific view influenced by social constructivism and symbolic interactionism. It aims for interpretative understanding of participants’ multiple perspectives and meanings. Whether a situation is a social problem or not depends on the meanings and judgments of the individual who commonly takes the problem for granted (Best, 2013). Hence, the individual’s height/weight growth is socio-culturally defined or constructed. The verbal interactions of parents with their adolescents, and other family members and relatives, socially construct realities of height/weight growth which by themselves create the structure and meanings of the concerns for parents. This socially collective reality is formed depending on time, place, and specific cultural context (Charmaz, 2006, 2014). This article presents the extracted codes and categories of such concerns in the framework of a comprehensive study.

### Setting

We conducted this study in Tehran, the capital of Iran. We recruited the first three eligible participants from a paediatric endocrine clinic at one general hospital affiliated to one of the universities of medical sciences. They shared their concerns regarding the height growth of their boys or girls based on their subjective ideals with an endocrinologist at this centre. The initial extracted codes and conceptual categories navigated us to recruit other key informants from the community and a primary school with the consideration of maximum theoretical sampling variation based on guide-lines in their experiences and socio-economic backgrounds from the north, centre, south-east and west zones of Tehran, in order to get a saturation on properties of related concepts (Table I).

**Table I.** Demographic characteristics of parents (n = 11).

Parenthood role	Adolescent’s age (Years) and sex	Adolescent’s education (Grade)	Birth order in the family in terms of adolescent age	Place of residence (Urban zone)	SES^*^ of family
Mother	Son, 17	11	Second	10	Moderate
Father	Son, 17	11	Second	10	Moderate
Mother	Daughter, 13	7	Second	10	Moderate
Mother	Daughter, 10	4	First	15	Moderate
Mother	Daughter, 13	7	Second	10	Moderate
Mother	Sons, 13 & 18	7, 11	Second, First	18	Low to Moderate
Mother	Son, 17	11	Third	18	Moderate
Father	Daughter, 12	6	Second	6	High
Mother	Daughter, 12	6	Second	6	High
Mother	Daughter, 12	6	Single Child	17	Moderate
Mother	Daughter, 14	8	First	1	High

* SES: Socio-Economic Status

### Sampling

The participants consisted of 11 parents (9 mothers, 2 fathers) of 10–18-year-old adolescents who resided in the different urban areas of the setting. The inclusion criteria were: (a) parent of an adolescent between the ages of 10 and 19; (b) having adequate time and information for the interviews; (c) the ability to speak in Persian; (d) the ability to communicate and reflect; e) to be interested in taking part in the study.

The participating parents were diverse in terms of their teens’ age, sex, and birth order in the family; place of residence; occupation; education; and self-reported socio-economic status of the family in terms of covering theoretical sampling framework (see part of the information in Table I). They were between 33 and 52 years of age. Six mothers were householders, two mothers were employees, and one mother was a teacher. One of the fathers was a retired employee and another was an employee. In terms of education, most of the mothers had a national diploma (ND). One of the mothers had secondary school education and three of them had bachelor and masters degrees.

In the recruitment process of participants, we initially selected the key informant parents using purposeful sampling to collect rich information and began the data analysis process. When we identified the emerging initial conceptual categories from data related to the first two participants, we continued theoretical sampling to recruit the next participants. To achieve the research aims according to the conceptual categories derived from the analysed data, the main researcher (PR) asked parents to introduce other eligible informant parents if possible. Moreover, a sixth-grade teacher introduced parents who had shared their concerns with her about their adolescents’ shorter height/smaller body build or higher weight compared to their peers. We performed theoretical sampling to collect enriched data based on extracted categories from previous data until the data saturation. Theoretical sampling forced the researchers to search the required data to develop the properties of conceptual categories and analysis gaps (Charmaz, 2006, 2014).

### Data collection

The interviewer (PR) conducted 11 intensive semi-structured qualitative interviews in person at the parents’ home/workplace or the main researcher’s workplace in a quiet place. Data collection and analysis iteratively took place between May 2015 and October 2016. The researcher conducted one interview with each participant, although she had two other short interviews with the first and ninth mother to clarify and explore the collected narratives. The interviews lasted 99 min on average and 1090 min in total.

Using the help of supervisors, the main researcher prepared an interview guide in line with the research purpose including a few open-ended and understandable questions in order to investigate the variability of the participants’ experiences which needed an open and interpretive response. Charmaz (2014) suggested that an interview guide is useful for novices and even experienced researchers to think through the kinds of questions that can help them attain their research objectives. In particular, novices require more structure to negotiate on a specific topic in a context. They should think about the sample questions and write a few open-ended questions for their study, although they might not follow their original questions. Having an interview guide with well-constructed open-ended questions and ready probes can increase researchers’ confidence and permit them to keep in mind how and when to ask questions in conversation. In the absence of a working guide, the interviewer may ask awkward, poorly timed, intrusive questions that can result in failure to elicit participants’ experiences in their own language. It is worth mentioning that the main researcher conducted the first interview in the presence of the first author, as one of the main supervisors in order to get his comments on conducting the next interviews.

With this approach, the main researcher determined the main lines of the interview, although she asked more detailed and specific questions based on parents’ narratives during the interview process to probe variations in their concerns. She occasionally glanced at her interview guide while conducting the interview. The researcher told all the participating parents that it is important for us to understand their experiences and concerns regarding the physical growth of adolescents through the interview as a negotiation and interaction between them to create data and conceptual frameworks. The interview process included the initial, intermediate, and ending questions (Charmaz, 2014). Echoing from Charmaz (2014), samples of the interview questions are presented in Box 1. The main researcher recorded all the interviews with a voice recorder and transcribed them verbatim for analysis.

### Data analysis

Inspired by Charmaz (2006, 2014), we conducted procedures of data collection and data analysis simultaneously. We transcribed the interviews verbatim and read the transcripts several times; as well as we performed the initial, and focused coding using gerunds to reach a sense of sequence in the participants’ statements. In the coding process, we categorized pieces of data with a conceptual label. We gave an initial code to each semantic unit of data (line-by-line coding) that was closely fitted with the data. The initial conceptual categories emerged using constant comparison of old and new data as well as initial codes. We sorted them based on their similarities and differences. We used the initial codes that appeared more frequently or were more significant to conceptualize focused codes with a view to properties of subcategories. The route of the analysis emerged from the parents’ narratives and the following coding. We used these genres of the coding in an emergent way for describing, and explaining concepts related to the parental concerns and their relationships. In this article, there is no claim on the development or production of a comprehensive theory to explain such concerns of parents. Also, in an endeavour to intensify the abstraction level of the ideas, the main researcher wrote memos in all phases of the data analysis process including coding, and categorizations. Memo-writing provides an opportunity for making a constant comparison between data, codes and categories, and for coherent articulating conjectures (Charmaz, 2006, 2014). We continued to collect and analyse data until no new concepts or properties of the categories emerged.

### Rigour and quality of results

The main criteria for valuing the rigour of data and results are credibility, originality, resonance, and usefulness (Charmaz, 2014). In order to improve the credibility, we took the following steps: we collected data with maximum variety by allocating adequate time for interviews along with constant interaction with parents; we established close contact with data by open coding and live codes; the participants confirmed the findings of each interview at the following interview; the main researcher discussed all the extracted concepts through the analysis process with all the authors and used the qualitative experts’ viewpoints (the supervisors as co-authors in this study [MAC and AV], and reviewers of the main researcher’s thesis); and we analysed carefully the data. We attempted to ensure the criteria of originality of the data by developing new categories through conceptual explanation of the data. In order to achieve the criteria of resonance, we conducted frequent interviews to enrich the conceptual categories. For the criteria of usefulness, this study provided a description of the perceptions of parents’ concerns that could be a matter for future research and the expansion of pertinent knowledge.

### Ethical considerations

We obtained ethical approval for this study from the Institutional/Ethical Review Board on 5 April 2015 (Ref.: 1395.2834). The main researcher introduced herself, talked about the aims and method of the study, and gave parents the opportunity to discuss their participation with their spouses and children. All participants voluntarily signed the informed consent form. The main researcher ensured that she preserved confidentiality and anonymity, and the participants would have the freedom to withdraw from the study or to cease the interview at any time. She informed all the participants about the necessity of using a voice recorder and sometimes recording notes during interviews. All the participants permitted her to record their voices before the commencement of the interview. She emphasized that they could ask her to turn off the recorder at any time. She arranged the time and place of the interviews with those who agreed to participate.

## Findings

**Figure 1.**
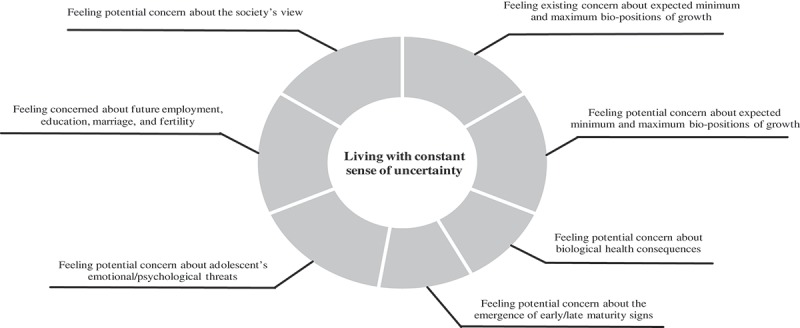
Main emerged category and subcategories.

As we demonstrated in Figure 1 and Table II, the concept of “Living with constant sense of uncertainty” emerged as a result of inferential leap of 7 initial conceptual categories and 62 initial codes; that is, the parents were occupied by thoughts of their adolescent’s height/weight growth. They had experienced concerns regarding their daughter’s/son’s physical growth with more attention to the body size or height/weight through the transition of adolescence. Such feelings created a sense of insecurity during the parents’ life, which can have emotional burdens and costs for the parents. We explained the following subcategories and exemplified each concept by using one parent’s direct quotes.

**Table II.** The properties of concepts relevant to parents’ concerns.

Initial codes	Initial conceptual categories	Focused code
Feeling concern of parents about continuous overweight and shorter height of their 13-year-old son	Feeling existing concern about expected minimum and maximum bio-positions of growth	**Living with constant sense of uncertainty**
Feeling concern of parents about shorter height of their daughter in comparison with her peers		
Feeling concern of father about shorter height of his daughter due to mother’s opinion		
Feeling concern of mother about shorter height and overweight of her second son		
Feeling concern of mother about lack of proportionality between weight and height of her second son		
Feeling concern of parents about overweight of their first and second sons		
Feeling concern of mother about more growth of breast in her first son (at the age of 14) and his small testicles (at the age of 8–9)		
Feeling concern of mother about lower weight gain of her son than his height growth.		
Feeling concern of father about shorter height and lower weight of his daughter relative to her age (10)		
Feeling concern of mother about overweight and taller height/larger physique of her daughter		
Feeling concern of mother about smaller body build of her son which can be a reason for being bullied by his friends	Feeling potential concern about expected minimum and maximum bio-positions of growth	
Feeling concern of parents about future overweight and difficulty in losing weight in their son		
Feeling concern of mother about future lower height growth of her daughter with possible diagnosis of an endocrinologist		
Feeling concern of mother about more height growth of her second daughter in comparison with the first one		
Feeling concern of mother about more future height growth of her daughter like her father concurrent with action to get taller		
Feeling concern of mother about her son inheriting his lower height growth from her relatives		
Feeling concern of mother about bisexuality of her son due to his having large breasts		
Continuing feeling concern of mother about large breasts of her son as she had not seen similar cases		
Feeling concern of mother about continuous lower weight of her son and not willing to eat		
Feeling concern of father about lower height and weight growth of his daughter over time		
Feeling concern of mother about possible overgrowth of her daughter’s height by playing volleyball		
Feeling concern of parents about possible adenoma and blindness due to diagnosed deficient growth hormone	Feeling potential concern about biological health consequences	
Feeling concern of parents about physical health consequences of their daughter/son due to overweight		
Feeling concern of mother about future weak body of her daughter due to her smaller body build and refusing to eat		
Feeling concern of mother about overweight and lack of activity in her daughter		
Feeling concern of mother about cessation of her daughter’s height growth and the emergence of maturity signs	Feeling potential concern about the emergence of early/late maturity signs	
Feeling concern of mother about earlier start of maturity/period and cessation of height growth in her daughter		
Feeling concern of mother about passing through maturity age, and cessation of height growth and continuity of overweight in her son		
Feeling concern of mother about earlier maturity of her daughter due her fast growth at the age of 8		
Feeling concern of mother about earlier maturity of her daughter with larger body build at the age of 5 and under physician supervision		
Feeling concern of mother about delayed period and continuation of height growth in her 14-year old daughter		
Feeling concern of mother about shorter height of her daughter and her feeling of incompleteness	Feeling potential concern about adolescent’s Emotional/psychological threats	
Feeling concern of mother about shorter height of her daughter and her future lack of self-confidence		
Feeling concern of mother about shorter height of her daughter and her future depression		
Feeling concern of mother about her daughter’s peers talking of her shorter height and her feeling of being defective		
Feeling concern of mother about lower weight of her son and his humiliation and being labelled as a drug addict among relatives		
Feeling concern of mother about smaller body build of her daughter and her future emotional problems due to friends’/spouse’s judgment		
Feeling concern of mother about larger body build and humiliation of her daughter at family gatherings by being reminded about not eating too much		
Feeling concern of mother about overweight and earlier period of her daughter and her future emotional problems		
Feeling concern of mother about her daughter’s emotional problems due to being labelled as fat by her future child		
Feeling concern of mother about her daughter’s emotional problems due to her larger body build and decrease of her efficacy		
Feeling concern of mother about her daughter being ashamed due to her larger body build and avoiding communities		
Feeling concern of parents about shorter height of their son and selecting his desirable job	Feeling concerned about future employment, education, marriage, and fertility	
Feeling concern of father about smaller body physique of his son and his ability to work		
Feeling concern of parents about shorter height of their son and spouse selection		
Not feeling concern of mother about shorter height of her daughter and choosing her favourite job		
Feeling concern of mother about shorter height of her daughter and success in future job		
Feeling concern of mother about shorter height of her daughter and continuing her studies with feeling of incompleteness		
Feeling concern of mother about smaller body build of her daughter and her weak body and fertility		
Feeling concern of parents about continuous obesity of son and his ability to work manually and make money		
Feeling concern of mother about continuous shorter height of her son and his ability to work manually		
Feeling concern of parents about smaller body build of their daughter and her intelligence, future job, education, and marriage		
Feeling concern of mother about her daughter's taller height and less attention to studying due to being made to sit at the back of the classroom		
Feeling concern of mother about overgrowth of weight/height of her daughter and her future marriage		
Feeling concern of mother about height/weight overgrowth/larger body build of her daughter and her fertility, marital commitment, and child care		
Feeling concern of mother about society’s opinion of shorter people	Feeling potential concern about society’s view	
Feeling concern of mother about continuous lower weight of her son and being labelled as drug addict by family		
Feeling concern of parents about continuous lower growth of weight/height of their daughter and being ridiculed by society		
Feeling concern of father about continuous lower height and weight growth of his daughter and future being eye-catching		
Feeling concern of mother about continuous overweight of her daughter and being ridiculed in society or her married life		
Feeling concern of mother about larger body build of her daughter and being labelled as fat/ugly body/heavy by society		
Feeling concern of mother about larger body build of her daughter and other expectation of her acting more wisely

### Feeling existing concern about expected minimum and maximum bio-positions of growth

The first subcategory showed that, according to the parents’ description, the dimensions of these concerns included minimum bio-positions of growth (e.g., shorter height, gaining less weight compared to height, smaller physical build, and maturity later than what the parents expected) and maximum bio-positions of growth (e.g., taller height, higher weight, bigger physical build, and maturity earlier than what the parents expected). Two parents explained it like this:

She was always smaller than usual. For several years, I noticed that when she was in school, or goes to her friends’ birthday parties, her peers were twice her size. Some examinations and tests have been done and we are worried that her height and weight are not proportionate to her age. (Father of a 12-year-old girl)

The problem we have is her weight. I think this is in our genes, although we have never had such a fat child in the family. (Mother of a 14-year-old girl)

Indeed, the parents assumed that the height/weight growth of their adolescents is different from the social and biomedical norms when they compared their children with their peers and the family members at a look, although they believed that the growth is genetic.

In a few cases, the parents were concerned about other aspects of growth related to the size of genital organs as one of the secondary sexual features. One mother stated her concern in this way:

His testicles were very small. I saw other boys’ testicles, and then I talked to my husband about it. (Mother of an 18-year-old boy)

This mother was concerned about the small size of her son’s testicles when he was 8 or 9 years of age; i.e., 1–2 years before the child’s adolescence. Her concern continued until she consulted a physician.

### Feeling potential concern about expected minimum and maximum bio-positions of growth

Nearly all interviewed parents in this study, based on their perceptions, were concerned that less or more growth of the height and/or weight of their adolescents may continue in the future. One mother explained her experience like this:

Her doctor said, imagine a plant in a pot that has not received any water for a long time, and it is suddenly given some water, it returns to life, but maybe she will not grow so well in the next months. I became a bit upset and worried. I thought she would continue getting taller, 4–5 cm, for the next six months which would have satisfied me. (Mother of a 13-year-old girl)

This mother thought that her daughter’s height would not increase as she anticipated in the next months, based on the explanation of her physician.

Another mother described her own experience of potential concern about her daughters’ height growth:

See; in a family, especially two sisters, I say that a few years later the younger sister will be taller than the elder one, which would be another story. For instance, I took my daughter to summer school; two sisters were there, one in 6th grade and the other was in 12th grade. I think the sixth-grade student was about 10–15 cm taller than her elder sister … then their mother said this is my elder daughter, she studies in 12th grade … I really did not know what to say (laughing). I said to myself that why is she so small? … At first look they are not matched in some ways. (Mother of a 10-year-old girl)

She thought that her elder daughter would have less height growth or smaller body build in comparison with her younger daughter. She believed that, especially between the two sisters, body size growth (with accentuation on height growth) in the elder sister should be more than the younger one. In other words, she imagined that the body size should correspond with age.

We found this type of concern about other aspects of growth regarding secondary sexual features:

My elder son’s breasts were like a young girl’s breasts. Men’s breasts are flat but his were swollen around 13–14 years during his puberty. I was really concerned. His breasts are still the same. I am still a bit worried, as I have not seen this form in men even in fat men. There is an androgynous person in our neighbourhood, so I asked the family of that person … I was constantly worried that my son might be androgynous. (Mother of an 18-year-old boy)

This mother felt concern about the excessive growth of her son’s breasts during early adolescence, and even after being ensured by the doctors she was still concerned. She regarded it as unusual.

### Feeling potential concern about biological health consequences

The analysis indicated that six parents were concerned about the possible consequences of biological health with the existence or continuation of smaller height growth and physical build, or heavier weight in their adolescents, as seen in this account from one mother:

The level of fat in the blood, diabetes, cardiovascular problems … all of these exist in her father’s family. One factor that motivated me was the effect of my obesity on my ovaries. Her aunt was obese and had the same problem. Obesity causes this problem, and Polycystic Ovarian Syndrome causes obesity. So I am worried. (Mother of a 14-year-old girl)

Based on their perceptions, parents who have adolescents with higher weight growth appeared to be more sensitive and stated their feelings strongly.

### Feeling potential concern about the emergence of early/late maturity signs

Six parents were concerned about the fact that the growth of their daughter/son in terms of height might stop or become slower when maturity begins. One of them said:

Well, for example when they talk about maturity in school, I get really worried. I thought about it for few days. What if her maturity begins at this age, what would I do? (Mother of a 10-year-old girl)

Another mother assumed that the current height (160 cm) of her 13-year-old daughter is normal, but she would have preferred it to be 170 cm. She expressed her opinion in these words:

She had her first phlebotomy. I thought it might delay her menstrual cycle for a year to give her more chance to grow taller. One year is enough time for her to grow taller. We did the second phlebotomy and then her menstrual cycle began. (Mother of a 13-year-old girl)

This parent stated that she had stopped growing in terms of height with the onset of her menstrual cycle. She felt that her daughter will probably have the same problem at the age of 13 due to her own experience.

The mother of a 12-year-old girl said that she was worried about possible earlier maturity at about 8 years, due to the fact that she thinks the growth of her daughter in terms of weight was more than usual. Another mother who had a 14-year-old daughter had a similar concern. The latter mother said:

First we thought her excessive weight might trigger earlier maturity. Her physician was seeing her regularly and was ordering uterine and ovaries ultrasounds and some hormone tests in order to detect any sign of premature puberty. This issue has been reversed now that she is 14 years old and yet to see her period. I was worried about her height since it is not normal for a 14-year old girl to be 182 cm and I don’t know what to do if she gets even taller. (Mother of a 14-year-old girl)

She said her concern started when her daughter was only 5 years old, because of her larger body build. She was under constant monitoring of a physician, until she became 7 years of age, for any sign of earlier maturity. This concern changed since her daughter did not show any menstrual cycle signs at the age of 14. She thought her daughter was getting taller and taller due to delay in her menstruation.

### Feeling potential concern about an adolescent’s emotional/psychological threats

Seven parents were worried that the smaller build, heavier weight, and earlier maturity of their daughter/son than the parents expected would impair their emotional health, which may also affect their self-confidence and may even lead to depression, let-down, stigmatization, emotional trauma, and shamefulness. One of the mothers expressed her feelings about her daughter, who has a smaller body build, like this:

Well, when you see you lack something as compared to the rest of the society, it affects you. She may get depressed and lose her self-confidence. (Mother of a 10-year-old girl)

She thought that her adolescent may feel abnormal when compared with her peers, due to her shorter height which may lead to low self-confidence and depression.

The following account shows the perception of a mother whose daughter had earlier maturity:

I had read that some children start their period in the third grade. This will affect their mental health … Kids at this age should go and play, but these children are ashamed to do so, it is scary for them. That is why I was so worried. I did not want her to have to deal with adult issues at her age. (Mother of a 12-year-old girl)

This parent thought that earlier onset of menstruation due to body weight overgrowth troubles her daughter’s mental health. They may be afraid of this and feel embarrassed among their friends due to their younger age.

### Feeling concerned about future employment, education, marriage, and fertility

About half of the parents were concerned about the future occupation of their adolescent children and the other half were concerned about their future marriage and spouse selection because of their more or less height/weight growth than the usual for adolescents based on their expectations. A parent declared:

Yeah, finally my child wants to go find a job, they look at his height and body physique. Finally, he should be able to do his job. (Father of a 17-year-old son)

Another parent stated her opinion in this way:

I don’t know (laughing), I feel these are maternal thoughts. Well, I feel if my son for example has the same height (shorter height) and wants a wife who is pretty with tall height in the future, the woman would not want to marry a chubby short man. (Mother of a 17-year-old son)

Meanwhile, a mother pointed out that her daughter with shorter height has selected her future job and, as a result of this, she had no obsession about her future career.

Three parents were concerned about the future education of their daughters. One of the parents described his concern in this way:

The issue is intelligence and memory if she eventually wants to go to university or get a job; this will affect her education and occupation. This will also affect her marriage and life in the future. (Father of a 12-year-old girl)

They thought about lower height growth and possible feeling of defect, smaller body size and less intelligence, or less focus of the adolescent with height overgrowth, and as a result of this going to sit at the back of the classroom.

Two mothers were worried about the smaller body size or extra weight of their daughters which may affect their physical weakness and/or performance. One of them expressed her sensitivity to the topic in this way:

If she loses weight in the next one or two years through some kind of diet, then the challenges she might face in the future will reduce. When she eventually wants to get married, give birth, and deal with marital life issues, I am afraid that she may not be able to due to these things. (Mother of a 12-year-old girl)

They believed that smaller body physique or more weight growth may affect their body capability of getting pregnant, giving birth, taking care of their baby, and dealing with married life.

### Feeling potential concern about the society’s view

Eight parents were concerned about the effects of stigmatization by society on their adolescent. One parent declared her view of the matter:

For example, once someone called her “a big girl” in the park (e.g., be careful about that big girl, she might crush you) or when we are in family gatherings (e.g., look how big that girl is. What a pity, she has a beautiful face but her body is ugly). (Mother of 14-year-old girl)

The parents took labelling by other individuals for granted, because nobody wants to be eye-catching. They meant that their daughter/son can encounter labels such as “short/too tall”, “skinny/fat”, “unfit”, or “addicted” due to lower or higher height/weight growth, or larger body size. They believed that people look for normal height and weight and nobody wants to be eye-catching.

## Discussion

**Figure 2.**
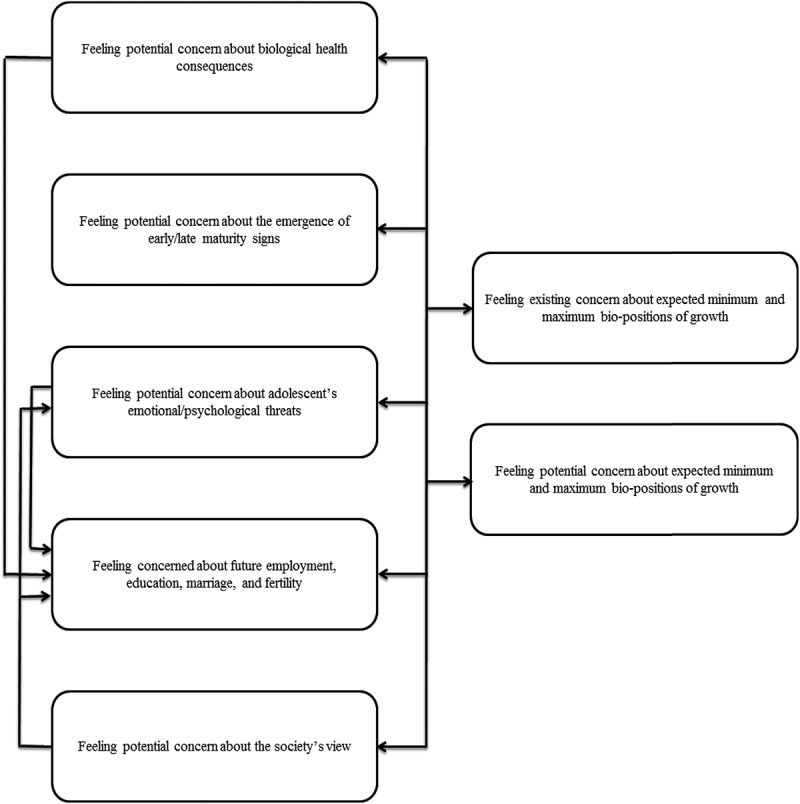
The connection among the emerged concepts regarding parents’ concerns.

This study provides fresh insights on concepts that have to do with parents’ concerns regarding the growth characteristics of their adolescents. From the response of the interviewees, it was observed that the parents expected the growth of their children’s height/weight to be compatible with that of their sibling and/or other peers all through adolescence. Also, most parents in this study assumed that the height (growth) of their children ought to be proportional with their weight (growth). The difference between their adolescents' growth and defined subjective and objective (biomedical) norms of growth from the angle of body size and rarely secondary sexual features was a motive for the emergence of parents' worry with it, and gloomy outlook and uncertainty toward the unknown future of well-being of their adolescents, while this may be nothing to worry about and their understanding of this issue as deviation from normal condition. These findings are in agreement with previous studies showing that parents, particularly mothers, feel concerned about the shorter height and higher weight of their adolescents, and they want them to adjust (Lampard et al., 2008; Lee et al., 2009; Pope et al., 2014; Styles, Meier, Sutherland, & Campbell, 2007; Balen et al., 2005). In line with the parents’ views, the present study suggests that such parents’ existing and potential concerns about their adolescents’ bio-positions of growth can be affected by the following other potential concerns extracted (Figure 2).

In this study, more than half the parents thought that shorter height, smaller body build, and higher weight of their teenagers may endanger their present and/or future biological/physical health. This finding supports the findings of Pope et al. (2014) who have shown some maternal caregivers aiding their adolescent to manage weight since they did not desire their girls to end up having hypertension, diabetes, or other problems. In the present study, these parents believed that these health challenges may consequently have an unpleasant effect on their adolescent’s ability to do manual work and to make money/revenue, and also on their future job success. On the other hand, there were parents who considered that their daughters with smaller body physique and possible body weakness or higher weight would have less physical ability for continuing education, becoming pregnancy, childbearing, and even caring for their children.

One major topic that needs consideration is public discourses centred on biomedical construction of appropriate body size in a community and its association with good health, as reported by others (Best, 2013; Conrad, Macki, & Mehrota, 2010; Rosenberg, 2009; Wills et al., 2006). The present study suggests that parents may consider their adolescents with height and/or weight outside the medical criteria as unhealthy by accepting this view. This issue can be reflected by parents, concerning their bio-positions of growth. Therefore, as reported by other researchers (Puhl, Himmelstein et al., 2017; Wills et al., 2006; Styles et al., 2007), it seems that health-care experts should apply impartial terms like “weight” or “height” instead of “obese”, “heavy weight/body build”, “short”, and “small body build” when discussing with parents or adolescents. This needs to be explored further.

In the present study, more than half of the parents reported their concern about the emergence of earlier or later signs of maturity in their daughter/son than they had estimated based on their imagination. Life experiences of these parents showed that the speed of height growth gets slower with the onset of the first menstruation in a girl or the first pubertal event in a boy, as they believed. By considering similar approach, one of the mothers, who was concerned about her daughter’s higher height, thought her growth in terms of height would stop if she got her first period as soon as possible. Some evidence revealed that earlier puberty than the social average is another concern for families/physicians, which may lead to an increase in bone growth and premature closure of the growth plates and make the adolescent shorter as compared to other peers (Yousefi et al., 2013). This issue has not been endorsed by some researchers and it is believed that there is a physiological compensatory mechanism between growth before and during puberty (Pantsiotou, 2008). The findings of the present study recommend that parents should be assured that there is no evidence that the timing of pubertal onset is a predictive factor of adult height, as reported in another study (Yousefi et al., 2013). Moreover, there were two mothers who believed that the excessive weight of their daughters may bring earlier maturity for them, which might be risky for the adolescent’s emotional health, *per se*. The results in the studies by Solorzano and McCartney (2010) and Chen et al. (2017) support this belief, although more research is needed to clarify these effects, as suggested by Solorzano and McCartney (2010).

From the findings of the present study, most parents complained that their daughter/son with smaller body physique, lower weight gain than height, or higher weight as compared to the average population may suffer emotional harm when entering the community (e.g., feeling inadequate, low self-confidence, mood irritation, depression, being labelled, and feeling humiliated by others’ teasing). This impression is supported by a few studies (Deaton & Arora, 2009; Krayer, Ingledew, & Iphofen, 2008; Rees et al., 2009; Visser-van Balen, 2005). Contrary to this, the findings by Kelnar (2012) and Bjerkeset et al. (2008) have not shown any association between shorter stature in adolescents/adults and their mental capacities. This perception is another topic that needs to be reflected on. Such potential concerns can depend on reactions of significant others or society to the characteristics of one person. Echoing a constructivists’ approach (Best, 2013; Charmaz, 2014; Ong, 2012), the reality of shorter/taller height or higher/lighter weight makes sense in the context of community. Accordingly, the present study suggests bilateral perceptual and verbal interactions between parents and adolescents and other family/community members construct parents’ outlook or values about their adolescent’s bio-positions of growth. Parents’ understanding of taller height as a value in a community forms their ideas, feelings, thoughts, and obsessions regarding the bio-positions of growth on their daughter/son.

As stated in some studies (Lampard et al., 2008; Watt & Ricciardelli, 2012; Zokaei, 2009), parental concerns and their frequent height-/weight-based compliments to or criticisms of their adolescent can contribute a lot to their adolescent’s negative feelings and inappropriate perception of their body shape and constant comparison of themselves to others. In some contexts, the concerns of parents about the excessive weight of their child/teenager can be connected with their adolescents’ body-image perceptions (Garousi, 2014; Pope et al., 2014; Spiller, 2009; Wills et al., 2006). On occasion, some obese adolescents blame their excess weight on themselves (Lewis et al., 2011; Lindelof et al., 2010) and feel happy when they lose weight, even though they think less about obesity-related health consequences (medicalization) (Lindelof et al., 2010; Spiller, 2009; Wills et al., 2006).

In addition, in the present study, the parents’ perceptions showed that their potential concerns about society’s view can affect their potential concerns about their adolescent’s emotional threats. For example, some of them were concerned about the adverse emotional effect of significant others’/relatives’ judgement on the larger/smaller body build of their daughter or the lower weight gain than height of their son. In fact, another main topic is that the existing stereotypes/schema of individuals in a community concerning lower or higher height/weight growth might lead to parents’ concern about the fact that their adolescents may face such views. For example, many parents in the present study noted their potential worries about height/weight making their child or adolescent socially conspicuous, being ridiculed, and labelled as “addicted”, “obese”, and “heavy”. Recent studies support these findings (Lewis et al., 2011; Puhl, Himmelstein, et al., 2017; Sand et al., 2015). These studies showed that the effects of the stigma or labelling of obesity and weight-based bullying are associated with low self-esteem/self-confidence and general/social well-being, discrimination, depression, anxiety, reduced social status/support/identity, problems in communicating with others, and loneliness. Similarly, Zokaei (2009) and Watt and Ricciardelli (2012) in their studies showed that some boys tend to gain more weight, arguing that with a stronger appearance they will be safe from others’ bullying.

Another topic that can be suggested as an influencing factor on individuals’ beliefs and judgement is public definitions and dialogues regarding height-/weight-based body fitness, which also seems to create an important aspect of body-image among some young men/women in some cultures. Some researchers are in agreement with this belief. The young people expressed their views by comparing their appearance with society’s ideal and non-Asian individuals, which is a concern in the sociocultural context of Asia (Charvadeh & Kermani, 2010; Wills et al., 2006; Zokaei, 2009).

The findings of the present study suggest that the parents’ perception of socially defined height/weight/body size standards in getting some jobs and selecting a spouse was a factor in their concern regarding the future employment and marriage prospects of their adolescents. For instance, some parents stated that some jobs (e.g., pilot job, military job) need taller height or larger body physique. In addition, a few mothers believed that smaller/larger body size of their daughter could have an impact on her future marriage and marital relationship. These findings have been confirmed by some researchers (Case & Paxson, 2008; Charvadeh & Kermani, 2010; Magnusson et al., 2005; Rosenberg, 2009; Samaras, 2012; Zokaei, 2009). While these studies showed that in some communities taller height has a positive correlation with an individual’s performance, success in revenue and marriage, and greater intelligence, Samaras (2012) presented that the acceptance of taller height is a mistaken idea in other settings. Therefore, it seems that parents need to reflect on these views/ideas and its effects on constructing their obsessions *per se*.

As a result, this qualitative study suggests further studies with qualitative and quantitative approaches to explore the viewpoints/concerns of both parents and adolescents on all aspects of children’s and adolescents’ bio-positions of growth. It is important that the process of formation of parents’ and adolescents’ concerns in different sociocultural contexts is evaluated and explained in order to be effectively used in the field of adolescents’ RSH.

## Conclusion

Conclusively, the interviewed parents were constantly worried about their adolescents’ growth bio-positions despite their age and sex, although the mothers were more preoccupied with this issue than the fathers were. This was the case for all interviewed parents, whether they had a higher or lower education, were employed or a householder, which part of the city they lived in, and whether they belonged to low-to-moderate-income, moderate-income, or high-income families. Parents were concerned about the fact that their adolescent will probably face undesirable physical, mental, and social health consequences as a result of a different growth or body appearance/size from their own and the public’s perceptions/expectations.

In the sociocultural context of Iran, valuable concepts were obtained by conducting in-depth interviews to describe the properties of parents’ concerns and the relationships between them regarding their adolescents’ characteristic of growth. In particular, we adhered to the criterion of maximum variation in recruiting the parents who participated. However, this study had some limitations. First of all, the findings may not be generalizable to all parents in other cultural contexts and settings owing to the nature of qualitative studies. Nevertheless, the main researcher tried to describe and interpret the parents’ intensive and substantial experiences from different areas with various socio-economic levels by developing her skills for conducting the interviews, in creating an interactional climate, and in encouraging the parents to talk. Secondly, parents who participated voluntarily through our analysis process may have more strong feelings about their teenagers’ growth characteristics or height/weight growth. Such feelings might be different between parents based on their personal views and perceptions (Lampard et al., 2008).

This study can be important in developing effective initiatives/programmes for adolescents’ RSH in Iran with a multidimensional view by RSH policymakers, psychologists, medical anthropologists/sociologists, and school authorities. By understanding the properties of these concerns, physicians/health-care providers should be informed about the influencing factors on such concerns and practically help parents to deal with them and the possible related pressures to reduce the burden of adolescents’ socio-psychological health problems. Parents should have an idea of the different rapidity of height/weight growth and maturity changes in adolescence and that being “normal” does not mean “average” or “common”. These findings can also be published in popular and scientific-professional peer-reviewed journals.

## The positionality of the researchers

This research was the result of teamwork and all the authors contributed to the study design, conductance, and editing of the article. In particular, PR, the main researcher and second author of the manuscript, with an MSc degree in maternal and child health, has clinical experience of about 13 years in monitoring children’s growth in comprehensive health service centres with students of midwifery and school health instructors, and also discussing with parents about their children’s weight/height growth. PR has repeatedly witnessed the sensitivity of parents, especially mothers, about the height and weight growth of their children. When she ask about the reason for this, most of them want to have children with a weight and height acceptable to their society and expectations. Therefore, they compare the height and weight growth of their children with those of other peers among their relatives and friends. It seems that this concern of parents can continue until adolescence, since PR, as the mother of an adolescent, has met some mothers in schools and educational centres who were concerned about less height growth, more weight growth, and smaller body build of their adolescents in comparison with their peers. They tried to find solutions for their worry by interacting with other mothers. Accordingly, PR has always had this question: in our sociocultural context, what are the insights, perceptions, feelings, and experiences of parents from different classes of families about the physical growth of their adolescents? And how can it be constructed via interactions between parents, physicians, adolescents, and peers in the social life?

Hence, in cooperation with the other co-authors of this manuscript in the field of health/medicine and social sciences, PR conducted a qualitative approach-based study (in the form of a PhD thesis in the field of reproductive health) for better understanding on the reality or phenomenon of parents’ concerns about their adolescents’ weight/height growth in their society, and its features/dimensions. Reproductive health experts, along with other health-sector experts, could play a crucial role in the management of these concerns and the potential enhancement of the sexual and reproductive health of adolescents, due to their close relationship with parents (particularly mothers), regarding their adolescent’s physical growth, by understanding parents’ concerns and that how they are constructed. Management of parents’ mental image of their adolescent’s weight/height growth and their reaction to that could provide a supportive atmosphere in the house where the adolescent’s feelings about his/her body will develop, which can prevent undesirable and unexpected consequences. Especially, that their adolescents might face higher pressure or challenges about having a suitable body shape and size as they grow up and enter the larger sociocultural environment.

## Figures and Tables

**Box 1.** A sample of the open-ended questions in our interview guide.

*Initial open-ended questions* Could you please tell me why you decided to take your adolescent to the endocrine clinic or physician? May I ask what made you interested in your adolescent’s height/weight growth? Could I ask when you noticed your daughter’s/son’s height/weight growth for the first time? *Intermediate questions* Would you tell me how you feel about your daughter’s/son’s height/weight growth? Could you tell me what you think about your son’s/daughter’s height/weight growth? Could I ask you to describe your thoughts about your son’s/daughter’s height/weight growth? *Ending question to summarize* Tell me how your feelings about your daughter’s/son’s physical/height growth might change from the time she/he entered adolescence and started to grow? Do you have something else you think I should know to understand? Is there anything you would like to ask me? Based on the statements to clarify them, further questions were asked such as: Could you give me some examples? You said that—may you explain more? Would you tell me what it means?
